# Efficient Limb Range of Motion Analysis from a Monocular Camera for Edge Devices †

**DOI:** 10.3390/s25030627

**Published:** 2025-01-22

**Authors:** Xuke Yan, Linxi Zhang, Bo Liu, Guangzhi Qu

**Affiliations:** 1 Department of Computer Science and Engineering, Oakland University, Rochester, MI 48309, USA; 2 Department of Computer Science, Central Michigan University, Mount Pleasant, MI 48858, USA; zhang15l@cmich.edu; 3 School of Mathematical and Computational Sciences, Massey University, Auckland 0632, New Zealand; b.liu@massey.ac.nz

**Keywords:** joint range of motion, pose estimation, fast deep learning model, edge device, clinical assessment, RGB camera

## Abstract

Traditional limb kinematic analysis relies on manual goniometer measurements. With computer vision advancements, integrating RGB cameras can minimize manual labor. Although deep learning-based cameras aim to offer the same ease as manual goniometers, previous approaches have prioritized accuracy over efficiency and cost on PC-based devices. Nevertheless, healthcare providers require a high-performance, low-cost, camera-based tool for assessing upper and lower limb range of motion (ROM). To address this, we propose a lightweight, fast, deep learning model to estimate a human pose and utilize predicted joints for limb ROM measurement. Furthermore, the proposed model is optimized for deployment on resource-constrained edge devices, balancing accuracy and the benefits of edge computing like cost-effectiveness and localized data processing. Our model uses a compact neural network architecture with 8-bit quantized parameters for enhanced memory efficiency and reduced latency. Evaluated on various upper and lower limb tasks, it runs 4.1 times faster and is 15.5 times smaller than a state-of-the-art model, achieving satisfactory ROM measurement accuracy and agreement with a goniometer. We also conduct an experiment on a Raspberry Pi, illustrating that the method can maintain accuracy while reducing equipment and energy costs. This result indicates the potential for deployment on other edge devices and provides the flexibility to adapt to various hardware environments, depending on diverse needs and resources.

## 1. Introduction

The conventional method for measuring a patient’s upper or lower range of motion (ROM) involves using a handheld goniometer by a surgeon or healthcare professional in a clinical setting for medical purposes, such as musculoskeletal health. This necessitates patient visits to clinics for postoperative ROM assessments after various procedures. A handheld goniometer is user-friendly for trained professionals and relatively inexpensive [1]. However, mastering its usage requires a learning curve, and achieving consistent results with a goniometer can be challenging even for the same professional [2]. In recent years, alternative approaches like deep learning-based cameras have been explored to provide ease and accessibility while improving ROM measurement consistency. Additionally, there is a growing trend toward remote medical care applications, including limb ROM assessments, instead of requiring patients to visit health centers or clinics. In this context, resource-constrained edge devices, such as Internet of Things (IoT) devices or mobile phones, are being increasingly employed to enable healthcare systems to efficiently and affordably support near-real-time applications when processing large amounts of data collected by sensors [3]. By providing remote access to patient data, such devices empower healthcare providers to make informed decisions and design personalized treatment plans while reducing operational costs. Moreover, processing data locally on these devices improves data privacy and reduces latency resulting from internet communication, making edge devices a compelling choice for healthcare applications [4].

To this end, this study aims to propose a deep learning-based method for human pose estimation and subsequent limb range of motion assessment. The model should be compact in size and have fast processing times while maintaining accuracy comparable to traditional goniometer measurements for limb ROM assessment. Furthermore, the proposed model should be deployable and executable on a regular desktop PC, which is widely available in health centers and clinics, as well as on resource-constrained and budget-friendly devices in cases of budget constraints or remote access requirements. Ultimately, the proposed model design and the benefits of edge computing work together to make joint ROM assessment more accessible, efficient, and adaptable, thereby improving patient care and outcomes in the field of musculoskeletal health.

To estimate limb joints and assess the range of motion, some studies use an RGBD (Red, Green, Blue, and Depth) camera, such as Microsoft Kinect [5,6]. A powerful computer and an RGBD camera are required to obtain the HPE (human pose estimation), but this hardware setup is way more expensive than a handheld goniometer. An alternative study uses an RGB camera and a generic image classification convolutional neural network (CNN) model named VGG16 to estimate a human pose. Nonetheless, VGG16’s accuracy is insufficient [7], and the approach is not tailored for resource-constrained devices. As a foundational and complex issue in computer vision [8], human pose estimation (HPE) has garnered significant interest in recent years. Among the contemporary state-of-the-art HPE networks is the hourglass model [9], which offers exceptional joint prediction accuracy. However, constructing smaller networks is not cost-effective due to the high number of channels per layer and increased training complexity. Moreover, models such as the hourglass, designed for HPE tasks, utilize all major joints from both the upper and lower human body to facilitate learning and prediction.

To address these challenges, we propose a lightweight and fast limb range of motion assessment method utilizing a CNN architecture named hourglass. We propose a reduced version of the full-size hourglass model by reducing the number of hourglass modules and converting float point parameters in the model to 8-bit precision. Those changes make the model size reduce about 16×. We evaluate the performance in terms of effectiveness and efficiency on a desktop CPU. We also consider deploying the application with a cost-effective solution for certain clinical requirements to meet needs, such as remote limb ROM assessment at home. To achieve that, we explore various model design options suitable for resource-constrained devices and implement the application on Raspberry Pi 4 (RPi4), a popular and affordable resource-constrained device. Unlike other edge device-based medical care solutions relying on cloud computing [10], our proposed model can locally be deployed and executed on RPi4. This local data processing can offer faster response times and more efficient ROM assessment and ensures sensitive information related to joint movements. And ROM is managed at the edge and can lead to better user privacy protection [11].

The reduced and quantized hourglass model (RQ-HG) employed in the data processing phase operates more efficiently and conserves memory space compared to the baseline model [9] while still maintaining approximately 93.6% of the joint prediction accuracy performance relative to the baseline. To assess the model’s comparability to a manual goniometer in conducting ROM evaluations, we devise targeted evaluation tasks that measure limb ROM, including limb functions like elbow extension/flexion and lower limb functions. We also experiment on a Raspberry Pi, a commonly used embedded prototype device, demonstrating that the method retains accuracy while cutting equipment and energy costs.

This work presents the following contributions:

- We present a method for assessing limb ROM, emphasizing the model’s efficiency. The proposed model achieves high accuracy and low overhead. We demonstrate that the model can be deployed and executed on both a desktop computer and a resource-constrained device, making it suitable for a wide range of limb ROM assessment scenarios.
- The proposed deep learning model is lightweight and efficient compared to one of the state-of-the-art CNN architectures. The reduced and quantized hourglass model (RQ-HG) operates 4.1 times faster and requires 15.5 times less memory space than the baseline model [9].
- We conduct comprehensive experiments to evaluate the effectiveness of our design. The experimental results indicate that the proposed method yields RMSE values ranging from 3.21° to 4.25° for upper and lower ROM measurements compared to those obtained using a manual goniometer and exhibits a high degree of agreement with traditional clinical ROM tool measurements.

This article is a revised and expanded version of a conference paper [12], presented at ICMLA, Bahamas, 2022.

Organization: This work is organized as follows. Section 2 reviews the related work on ROM tasks using traditional and deep learning methods. In Section 3, we present a method for lightweight and fast limb ROM assessment, which comprises three key phases: reduction of the hourglass model, quantization of the reduced model, and execution of the ROM assessment. Additionally, we demonstrate the implementation of the proposed method on a resource-constrained device, showcasing its adaptability and suitability for a wide range of hardware environments. Section 4 details the implementation and evaluation metrics of the proposed system. Finally, we draw conclusions and discuss potential improvements in Section 5.

## 2. Related Work

### 2.1. ROM Assessment Using Camera-Based Systems

#### 2.1.1. Non-Deep Learning Approach

We provide an overview of recent research on traditional 2D camera techniques without the use of deep learning. Meislin et al. [2] delve into the examination of elbow movement through static imagery. Their study contrasts findings from digit images and goniometers and juxtaposes photographic captures sourced from both surgeons and test subjects. It is worth noting that participants were photographed before any instructional demonstration, resulting in variations in the representation of angles. However, the findings reveal no discernible difference in measurements derived from surgeons or participants’ images. In a related vein, Santos et al. [13] explore the knee’s range of motion by comparing data from a standard goniometer and a mobile goniometric tool. Other researchers probe the dependability of smartphones in quantifying wrist action. Wagner et al. [1] confirm a strong consistency between images procured by laypeople and experts. Their research also highlights certain constraints, such as the requirement for patient familiarity with smartphones, potential challenges for elderly individuals, the inability to distinguish between active and passive motion due to missing equipment, and the critical role of safeguarding patient confidentiality as technology progresses.

#### 2.1.2. Deep Learning Approach

Smartphone camera methodologies highlighted previously do not harness deep learning to ascertain joint positions or automated image analysis to aid surgeons in their assessments. As deep learning-based HPE progresses [14,15], it can supersede conventional photographic techniques, potentially conserving both time and expertise when assessing ROM. The evolution of deep learning in computer vision now allows for the pinpointing of joints solely using an RGB camera by pre-established datasets. Yahya et al. [7] conduct an investigative comparison of shoulder joint angle estimations between an RGB camera rooted in machine learning and the Microsoft Kinect—an RGBD camera. Their findings suggest that deep learning approaches are on par and have potential applications like rehabilitation.

#### 2.1.3. Recent Techniques and Systems for ROM

Recent advancements in ROM assessment have been marked by the introduction of new techniques and systems, each showcasing unique strengths and confronting specific limitations.

DigitalROM, developed by Muaremi et al., utilizes the Microsoft Kinect 3D camera for shoulder ROM assessment with high accuracy [16]. Despite its efficacy, it faces challenges like sensitivity to environmental factors such as lighting and space, as well as potential inaccuracies in tracking complex movements or occlusions. Similarly, computer vision solutions like Kinetisense and Goniometer Pro have demonstrated success in assessing ROM in various joints [17]. These systems, while effective, may encounter issues with calibration accuracy and require specific positioning, with potential errors in joint angle estimations, especially in rapid or complex movements. The accuracy is also influenced by the quality of smartphone cameras and the precision of software algorithms.

Additionally, multisensor methods, as explored by Beshara et al. in their study combining kinematic and physiological sensors, provide a comprehensive evaluation of limb mobility [18]. Research by Yahya et al. on shoulder joint angle estimations using an RGB camera further highlights the advancements in deep learning applications in ROM assessment [7]. The BumbleBee2 stereo camera system, used for upper extremity workspace evaluation in patients with neuromuscular diseases [19], requires the attachment of markers to the body. This can be cumbersome and may not accurately reflect natural movement. Additionally, the system’s practicality is constrained by the need for specific camera calibration and environmental setup.

The proposed model in this study, optimized for edge computing, balances accuracy and efficiency, signifying a shift toward efficient, cost-effective models suitable for various healthcare settings. The integration of the IoT and edge computing in healthcare, as indicated in [20], underscores the significance of these technologies in modern medical systems.

### 2.2. Model Compression

Improving the efficiency of HPE is essential, especially in applications necessitating real-time response or deployment in resource-constrained environments [21]. Here is a deep dive into various strategies to optimize HPE models for efficiency:

Network Pruning and Quantization: Pruning [22] is a technique that removes less important neurons, weights, or connections within a neural network, effectively reducing the model size and computational demand without significant loss in accuracy. By identifying and eliminating redundant parameters, pruning allows for a more compact model while retaining the critical features necessary for performance.

In conjunction with pruning, quantization of neural networks [23] involves converting a model’s continuous-valued weights and activations into a discrete set of values, often represented with lower precision. This process maps full-precision values (e.g., 32-bit floating point) to a fixed set of levels, typically within a lower bit-width format, such as 1-bit or 8-bit integers.

From Nagel et al. [24], depending on many factors such as the requirement of accuracy, training effort, design complexity, and supported hardware, quantization can be further divided into two categories: (1) post-static/dynamic training, which quantizes an already-trained float model, and (2) quantization-aware training, which performs quantization during the training time and uses the reduced-bit model in the inference time. Quantization-aware training models generally achieve higher accuracy compared to post-quantization training approaches. Network quantization approaches can be used for tasks in environments with resource-constrained devices, such as [25], or tasks where efficiency matters [26]. For different applications, different methods can be selected based on their need and resources.

Knowledge Distillation: Knowledge Distillation [27] is a model compression technique where a smaller model, often referred to as the student, is trained to mimic the behavior of a larger model, known as the teacher. Instead of learning from the original ground truth labels, the student model is trained on the softer output distributions (probabilities) of the teacher model. This softer distribution, which might contain information about the relationships between different classes, can be more informative than the hard labels, allowing the student to achieve better performance than if it were trained directly on the ground truth. By distilling the knowledge from a large, accurate but computationally intensive HPE model (teacher) into a smaller, faster model (student), it is possible to retain much of the performance of the larger model while benefiting from the increased efficiency of the smaller one as presented by Zhang et al. [26].

Neural Architecture Search [NAS]: NAS [28] automates the selection of optimal neural network architectures for specific tasks. Using search strategies like reinforcement learning or evolutionary algorithms, NAS explores a predefined architectural space, evaluating and refining designs based on performance outcomes. In HPE application, NAS can efficiently discover architectures that balance accuracy and computational efficiency, making them ideal for real-time applications on resource-constrained devices. Tan [29] introduces an EfficientNet achieving unparalleled accuracy and efficiency with substantially fewer parameters. This method scales ConvNets uniformly across depth, width, and resolution, enhancing existing compact model performance.

### 2.3. Hourglass Model for HPE

**Figure 1:**
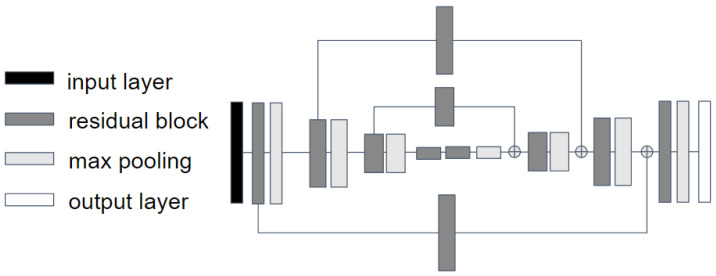
Single hourglass architecture [9].

**Figure 2:**
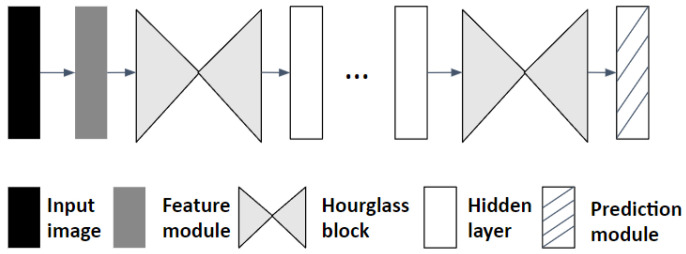
Stacked hourglass architecture [9].

Within the domain of deep learning, utilizing CNNs for image data processing has become a norm [21]. These CNNs are characterized by several layers—namely, convolutional layers, pooling layers, and upsampling layers—that collaborate to distill crucial visual features tailored for specific tasks. In our endeavor, we have adopted the stacked hourglass network, a leading-edge CNN blueprint for HPE undertakings, as our foundational model. This design incorporates a plethora of such layers, systematically organized to resemble an hourglass shape when graphically represented (refer to Figure 1). If we treat a singular hourglass as one module, the model’s schematic entails a consecutive arrangement of these hourglass modules, as illustrated in Figure 2. Each half of the hourglass structure consists of layered blocks. A standard block predominantly comprises a convolutional layer, a max-pooling layer, and ReLu activation functions.

Visualized in Figure 2, the hourglass design is a composition of hourglass modules, sequenced and interconnected. Paired with a loss function introduced between every module, this configuration facilitates intermittent supervision. Such a setup ensures that predictions stem from intermediate loss metrics, empowering the model to iteratively refine its predictions as the image transitions through the entirety of the stacked hourglass modules.

**Figure 3:**
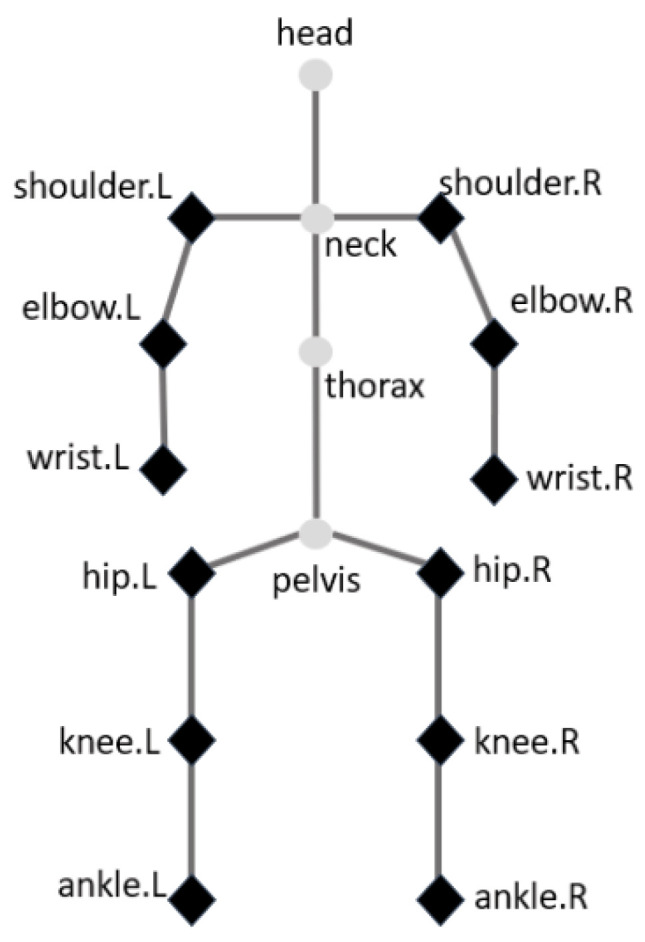
The designed model can estimate the joints of a human body for upper and lower joint movement, which are marked with dark black dots. L and R represent the left and right joints of the human body.

We use HPE algorithms to predict the upper and lower body joints shown in Figure 3. The joints that are learned and predicted in the HPE task are marked in dark black.

The outputs of HPE are the locations of the joints in an image, while ROM assessment needs to utilize the predicted joints and measure the moving range of an individual joint. The joint links and movement of those links are required to be further processed in the ROM task.

### 2.4. Medical Care with Edge Computing

The medical ecosystem has undergone significant evolution with rapid advancements in smart medical devices. Moreover, the progress of communication technologies has transformed various medical services into accessible systems and remote distance applications. The modern Internet of Things (IoT) implementation in medical systems has tremendously impacted public life and the healthcare industry [4,10]. While response time, availability, security, and privacy remain critical issues in cloud-based systems [4,10], edge computing serves as a key component of the IoT architecture, taking place directly on devices to which sensors are connected or on gateway devices physically close to the sensors. Examples of edge nodes include wearable devices such as smartphones and smartwatches and embedded systems like single-board computers and microcontrollers. Numerous studies [17,30,31,32] have demonstrated the effectiveness of resource-constrained edge devices such as RPi4, Arduino, or ARM-based microcontrollers in various medical care scenarios.

The research on limb range of motion assessment has seen significant advancements due to the integration of computer vision and deep learning techniques. Traditional approaches primarily relied on manual measurements using goniometers, which, while reliable, are labor-intensive and subject to human error. Recent studies have explored the use of advanced sensors and computer vision technologies to automate and improve the accuracy of these assessments. For instance, a multisensor method combining kinematic and physiological sensors like Kinect, inertial measurement units (IMUs), and surface electromyogram (sEMG) has been proposed. This method enhances the accuracy of joint movement classification and provides a comprehensive evaluation of muscle strength and limb mobility. These approaches represent a shift from manual to automated, sensor-based assessments, underscoring the evolving nature of kinematic analysis in healthcare and rehabilitation.

## 3. Methodology

**Figure 4:**
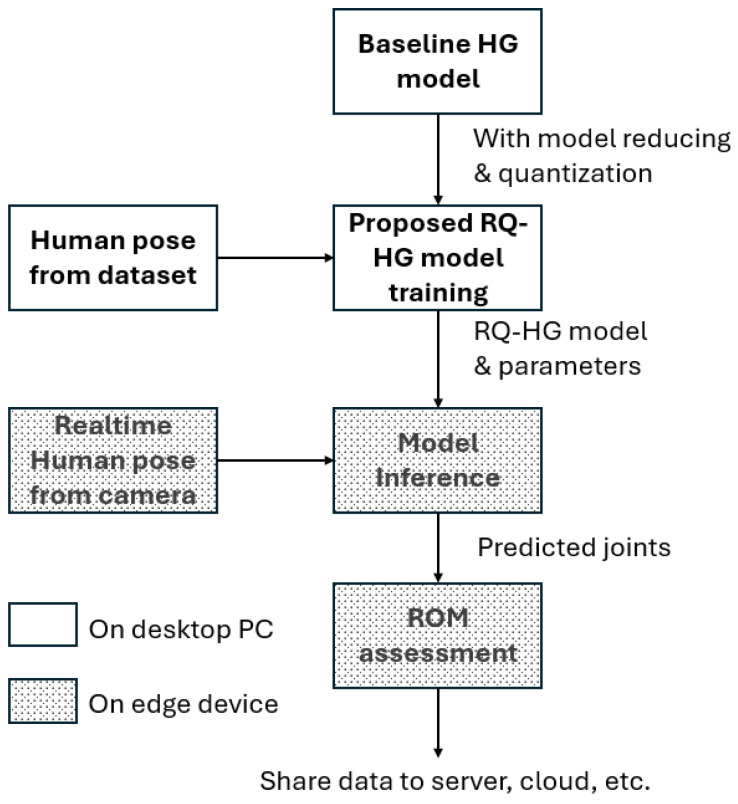
Workflow of the proposed deep learning-based limb ROM assessment system. The system consists of three phases: (1) reducing the baseline CNN architecture and quantizing parameters during model training, performed on a desktop PC; (2) real-time human pose estimation using 2D images captured by a cost-effective RGB camera; and (3) ROM assessment from predicted joint positions. Model inference and ROM assessment are deployed on resource-constrained edge devices, enabling efficient computation and adaptability. The results can be shared to servers, cloud platforms, or other endpoints for further use.

This section presents a deep learning-based limb ROM assessment system consisting of three phases using 2D images. The proposed model consists of three main phases: reducing the baseline CNN architecture, quantizing floating-point parameters to 8-bit, and measuring the ROM from the predicted joints. By utilizing a reduced stacked hourglass model and applying quantization-aware training, the method achieves faster computation and lower resource requirements, making it suitable for deployment on resource-constrained edge devices. This approach aims to improve the accessibility, efficiency, and adaptability of ROM assessments in musculoskeletal health, ultimately enhancing patient care and outcomes. An overview of the proposed system’s workflow is illustrated in Figure 4, which demonstrates that the model training phase occurs on a desktop PC, while image capturing and model inference can be performed on a resource-constrained edge device. This flexibility allows for efficient ROM assessment, adapting to various hardware environments to meet the diverse needs of musculoskeletal health evaluations.

The model’s design is based on a CNN-derived hourglass architecture and applies reduction and quantization techniques to make it lightweight and fast, which are essential factors for suitability in edge computing. This is due to the limited computational resources available on edge devices compared to regular desktop computers. Subsequently, we deploy the proposed model on a low-power, ARM architecture-based edge device and provide the model with input from a cost-effective 2D RGB camera.

### 3.1. Model Design

Deploying a CNN with a large number of channels and layers can result in a sub-optimal trade-off between the computational cost and the generalization capability. The stacked hourglass model [9], used as our base model, proposes such a CNN architecture with 8 hourglass modules where each has 9 residual blocks and 256 channels for each layer.

Zhang et al. [26] reveal that in the original hourglass model, the repeated hourglass modules and redundant channels can be reduced to half, 4 modules, while the compact architecture can still obtain enough generalization capability. Elhagry et al. [33] and Kim et al. [34] experiment with different module configurations and report the same conclusion that the original stacked hourglass model with 8 modules is redundant with poor cost-effectiveness. From the aforementioned works, even single or two stacked hourglass modules are capable of achieving 98% and 94% model performance, respectively. We summarize the performance comparison in Table 1 [26] from the same hourglass network structure but using different numbers of modules. The results are evaluated with the most common evaluation metric for the MPII dataset: mean PCKh@0.5. PCKh@0.5 is also used in the rest of this work, where PCK stands for the standard Percentage of Correct Keypoints, as our evaluation metric, which reports the percentage of detections that fall within a normalized distance of the ground truth. For the MPII dataset, the common practice is to set 0.5 of the head size, referred to as PCKh@0.5. If a predicted joint falls within this threshold, it is considered a successful prediction; otherwise, it is considered a failure.

The number of modules can be chosen depending on the computing budgets in practical use; in this work, we use a lightweight version of the original model which consists of 2 stacked hourglass modules. Compared to the original stacked hourglass architecture, the reduced model takes advantage of stacking multiple hourglass structures on top of each other, with only about 27% of (7/26) the number of parameters, which can significantly reduce the computational cost. Compared to the original design, the reduced one still retains 98% of (90.5/91.9) model performance.

#### 3.1.1. Model Quantization

The objective is to design a lightweight model to fast assess the range of motions. To accelerate inference and further reduce the model size, we apply quantization to the aforementioned model. Then, the model can perform computations and store parameters with lower bit widths instead of floating-point precision.

Our design quantifies the model with 8-bit for more compact model representation. Many quantization mechanisms are able to offer fast inference as well as lower computational costs. However, some of them cannot provide enough accuracy, such as post-training quantization. We make our reduced hourglass model quantized by quantization-aware training. We also achieve faster inference time because inference computational cost is saved while maintaining satisfactory accuracy performance without sacrificing too much inference accuracy.

Generally, quantization in neural networks introduces information loss that leads to a drop in accuracy compared to the floating-point model. To mitigate the accuracy decrease, we use quantization-aware training that considers such a loss during training.

Quantization can map a 32-bit precision value *x* to an 8-bit precision value $x_{8}$ in the range of $\left[\alpha_{8},\beta_{8}\right]$, where $\left(\alpha_{8},\beta_{8}\right)=\left(-2^{8-1},2^{8-1}-1\right)$, if the integer type is signed INT8. The quantization function [24] is as follows:

(1) $$f_{8}\left(x,s,z\right)=clip\left(round\left(\frac{1}{s}+z\right),\alpha_{8},\beta_{8}\right),$$

where *s* and *z* are the scales and zero point.

The de-quantization function is as follows:

(2) $$f_{d}\left(x_{8},s,z\right)=s\left(x_{8}-z\right),$$

and the information loss $\Delta_{x}$, due to quantization, can be calculated as follows:

(3) $$\Delta_{x}=x-f_{d}\left(f_{q}\left(x,s_{x},z_{x}\right),s_{x},z_{x}\right)),$$

By taking this loss into account during training, our quantized model is able to achieve satisfying accuracy during inference. In our reduced hourglass network, all the activations and weights are variables during the training. In the R-HG model from the previous phase, for the convolution, max-pooling, and ReLu activation layers, we add a quantization and a de-quantization layer for each layer.

In the training process, all the activations and weights are still floating points. For the forward propagation, the data are quantized and immediately de-quantized to add the information loss caused by quantization, which is similar to what might be encountered during quantized inference. For the backpropagation, there is a problem with the quantization-aware training because quantization and de-quantization layers are not differentiable. The derivative approximation of straight-through estimation (STE) can solve this issue [35].

(4) $$\frac{\partial \hat{x}}{\partial x}=\left\{\begin{array}{ccc} 0 & \mathrm{for} & x\geq \beta \\ 1 & \mathrm{for} & \alpha <x<\beta \\ 0 & \mathrm{for} & x\leq \alpha \end{array}\right.$$

Considering the derivative approximation of STE, the quantization and de-quantization function are treated as the same in the range [α,β] with result 1 and a constant function outside the range with result 0.

After the training process, we obtain the quantized model for inference. Since all the computations can be executed with 8-bit integer operations, the inference performance can be much faster than counterpart models with floating-point operations.

#### 3.1.2. ROM Measurement

Range of motion is the task of measuring the distance and direction a joint can move to its full potential. From the previous human pose estimation algorithm, we are able to estimate the joint position in images. There are upper and lower limb-related joints that can be obtained from our model in Figure 3, left/right shoulder, elbow, and wrist, etc., which are marked in dark black in Figure 3 with the predicted (x, y) coordinates for each. To measure the ROM for a functional task, such as elbow extension and flexion, we can calculate angles in geometry using Equation (5), where ${\bar{v}}_{s}$ and ${\bar{v}}_{e}$ represent a starting and ending position, respectively, for an elbow-to-wrist vector, and θ is the range of motion that an elbow rotates from an extension position to a flexion position.

(5) $$\theta =cos^{-1}\left(\frac{{\bar{v}}_{s}\;\cdot \;{\bar{v}}_{e}}{∥{\bar{v}}_{s}∥∥{\bar{v}}_{e}∥}\right)$$

### 3.2. Execution on Resource-Constrained Edge Devices

#### 3.2.1. Data Captured from Camera

Joint range of motion assessment using 2D imagery has been extensively explored in prior studies. In [1,2], the authors utilize smartphones to capture images of patients instructed to move or rotate their arms and elbows, enabling the camera sensor to document the movement and range of motion. Researchers subsequently analyze the captured images on a desktop PC, manually delineating lines between joints to measure the range of motion for shoulders, elbows, wrists, hips, and knees. Similar studies [13,36] have also demonstrated the capability of camera sensors to measure lower limb ROM. All investigations employing camera technology for joint ROM assessment have exhibited accuracies comparable to handheld goniometer measurements with some manual intervention (identifying joints from the image and measuring the ROM).

The proposed system optimizes the input size required by neural networks during the data processing stage, thereby minimizing the processing workload on edge devices. In our experiments and analysis, we found that using 360p resolution for input images strikes an ideal balance. This resolution allows for real-time video recording on devices and is well suited for our compact convolutional neural network. The choice of 360p ensures efficient processing without compromising the ability to effectively perform tasks, such as joint identification and motion analysis.

#### 3.2.2. Model Inference on Device

CNN architectures excel at approximating complex and non-linear mapping functions from arbitrary person images to joint locations, even in the presence of unconstrained human body appearance, varying viewing conditions, and background noise [9,37]. However, the advantages of model performance come at the cost of training and deploying resource-intensive networks with large depth and width. This leads to inefficient model inference, requiring a per-image computing cost at tens of floating-point operations (FLOPs) and poor scalability, particularly on resource-constrained edge devices [26].

In Section 4.3, we investigate methods including reducing model design and compression techniques to address the challenges associated with deploying deep learning models on resource-constrained devices. We experiment with reducing the number of hourglass modules from 8 to 1, ultimately settling on 2 modules to maintain an optimal balance between efficiency and effectiveness. Additionally, inspired by the parameters binarization approach on the human pose estimation model presented in [38], we observe a significant decrease in accuracy. To address this issue, we employ an 8-bit quantization-aware training method to convert the model’s parameters from 32-bit to 8-bit. This approach enables accelerated model execution while maintaining a lower degree of accuracy drop compared to the aforementioned binarization method. These optimizations result in a model size reduction to 1/16 of the original, enabling faster inference due to fewer parameters and reduced multiply–accumulate operations on floating-point numbers. Consequently, the proposed model is well suited for resource-constrained devices, making it deployable on a wide range of edge devices.

Edge computing has emerged as a promising solution for joint range of motion assessment applications due to its inherent advantages in terms of cost-effectiveness and localized data processing. Cost-effectiveness: Resource-constrained edge devices, characterized by their low equipment cost and low power consumption, make them an attractive option in various scenarios, particularly in remote or underserved areas with limited access to advanced healthcare infrastructure [39]. Medical professionals and patients can benefit from more accessible and convenient ROM assessment solutions. Localized data processing: Edge computing does not rely on a constant internet connection, allowing raw data to be securely stored and processed locally. This ensures sensitive information related to joint movements and ROM is managed at the edge and can lead to better user privacy protection [11]. Moreover, local data processing significantly reduces latency, resulting in faster response times and more efficient ROM assessment.

By leveraging the advantages of edge computing and the proposed model design, joint ROM assessment can be made more accessible, efficient, and adaptable, ultimately improving patient care and outcomes in the field of musculoskeletal health.

## 4. Experiments

In this section, we design four experiments to validate our design. (1) The first one is to evaluate the effectiveness of the proposed model for joint estimation. We compare joint prediction accuracy using the original hourglass network (HG), our reduced hourglass network (R-HG), and our quantized and reduced hourglass network (QR-HG). (2) The second experiment is to evaluate the efficiency of our model in terms of model size and inference time. (3) With the predicted joints, the last experiment measures the ROM for different limb functional tasks: elbow extension/flexion and shoulder extension/flexion. We calculate the root mean square error (RMSE) for the designed tasks using different measuring methods: a manual goniometer and the proposed camera with a deep learning algorithm. We also use a Bland–Altman analysis to define the limits of agreement between two measurement methods. (4) The fourth experiment is to discuss the cost-effectiveness of RPi4, taking into consideration equipment and energy costs and evaluating the model’s inference efficiency on the RPi4 platform.

In the following, we report the hardware and software used in this work and an input dataset used for training the designed model. We also report the ROM measurement procedures using a goniometer and a proposed 2D camera.

### 4.1. Experiments Setup

#### 4.1.1. Hardware and Software Setup

The experiments utilize a desktop CPU (AMD 3700x) and GPU (Nvidia RTX 2700 Super) to train the R-HG network, as proposed in design phase I of the data processing subsystem. An AMD 3700x CPU is employed to retrain the R-HG using the quantization-aware technique to obtain the QR-HG, wherein the weight parameters are converted to 8-bit. Both phases are implemented using Python 3.8 and the Pytorch 1.5 library. For data acquisition and processing of the trained model, we use a RPi4 Model B and a Raspberry Pi Camera Module V2-8 Megapixel. As a comparison to the edge device, we employ a desktop CPU (AMD 3700x) to run the same model as deployed on the Raspberry Pi.

A 12-inch, 360-degree physical therapy goniometer was used to manually measure the ROM in the experiment (see Section 4.5).

#### 4.1.2. Training Dataset for Joint Estimation

In order to train and evaluate our joint estimation algorithm, we chose MPII datasets [40] as our input data. This dataset is widely used in the human pose estimation area. The MPII Human Pose dataset is a state-of-the-art benchmark for the evaluation of articulated human pose estimation. The dataset includes around 25K images containing over 40K people with annotated body joints. The images were systematically collected using an established taxonomy of everyday human activities. The MPII dataset includes 410 diverse human activities, such as recreational, occupational, and household tasks, captured from various viewpoints. These activities involve natural joint movements like bending, reaching, and stretching, which are essential for training our ROM assessment model. The dataset’s diversity and range of viewing angles enhance the model’s ability to generalize to real-world scenarios, ensuring accurate and robust joint mobility evaluation in clinical and everyday settings.

#### 4.1.3. Limb Functional Tasks

We have developed six limb functional tasks, each designed to assess the mobility of specific joints on both the left and right sides. The tasks focus on the elbow, shoulder, and hip and include the following:

Elbow Extension/Flexion: Extension: This movement involves straightening the elbow to decrease the angle between the forearm and the upper arm, typically reaching toward a straightened position. Flexion: This refers to bending the elbow to increase the angle, bringing the forearm closer to the upper arm.

Shoulder Adduction/Abduction: Adduction: This is movement of the shoulder that brings the arm closer to the body’s midline, typically lowering the arm to the side. Abduction: This is lifting the arm away from the body’s midline, usually moving the arm out to the side and up toward the head.

Hip Extension/Flexion: Extension: This involves moving the leg backward, away from the front of the body, which increases the angle between the thigh and the torso. Flexion: This is the act of bringing the thigh up toward the torso, decreasing the angle between the front of the thigh and the torso.

These functional tasks are designed with practical applications in mind, commonly utilized for the assessment of joint mobility in various real-world scenarios:

Elderly Individuals: The tasks can help evaluate joint mobility in elderly individuals for the early detection of musculoskeletal issues, such as arthritis or joint stiffness. By identifying a reduced ROM in specific joints, clinicians can intervene early with tailored physical therapy or medical treatments to maintain mobility and improve quality of life. Athletes: For athletes, these tasks can monitor joint performance and flexibility, aiding in injury prevention and performance optimization. For example, monitoring shoulder abduction and adduction can help identify potential strain or imbalance, which could lead to injuries such as rotator cuff tears. Rehabilitation Patients: The functional tasks are particularly useful for assessing rehabilitation progress in patients recovering from surgery or injuries. For example, monitoring hip extension and flexion can track recovery in patients who have undergone hip replacement surgery or are recovering from a sports-related hip injury. Regular assessments can guide adjustments in therapy programs to ensure effective recovery.

For each of these functional tasks, we capture two digital photographs: one for the initial position (start point) and another for the final position (endpoint) of the ROM assessment. In parallel, we adhere to clinical standard procedures [2,41] to measure the ROM of the elbow, shoulder, and hip using a goniometer. This method allows for the precise quantification of joint angles, providing reliable data for evaluating joint functionality.

#### 4.1.4. ROM Measurement Protocol

We enrolled two healthy participants (one male and one female), both aged between 30 and 40 years, with no known joint issues. The participants obtain 2D camera photographs of full elbow flexion and extension. Then, the proposed deep learning algorithm is used to predict the limb joints and measure the ROM. Meanwhile, as a comparison group, we obtain the goniometric measurement of the same ROM for the same participants. This process is repeated, and each participant obtained a set of 10 sets of 2D camera measurements and goniometer measurements.

### 4.2. Experiment I: Joint Estimation Effectiveness

**Figure 5:**
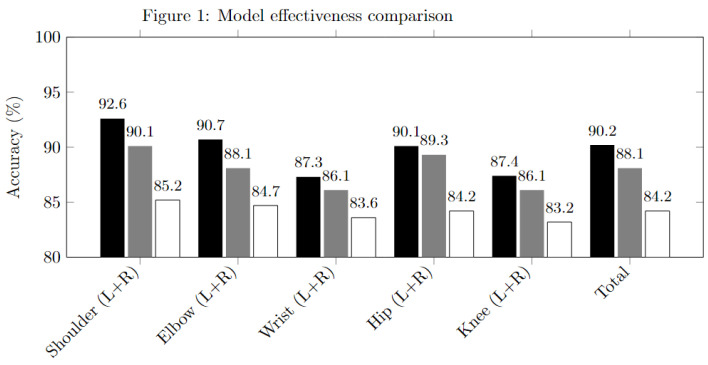
Model effectiveness comparison showing the accuracy (%) for different methods. Black bars represent the HG method, gray bars represent the R-HG method, and white bars represent the QR-HG method.

In this experiment, we demonstrate the effectiveness of the joint estimation algorithm in Figure 5. The baseline model from [9] is denoted as HG, while the module number reduced hourglass model is denoted as R-HG, and the quantization and reduction model is denoted as QR-HG.

We evaluate the accuracy performance of the shoulders, elbows, and wrists, as well as the accuracy of the limb joints. We choose the standard Percentage of Correct Keypoints (PCKs) as our evaluation metric, which reports the percentage of detections that fall within a normalized distance of the ground truth. For the MPII dataset, the common practice is to set 0.5 of the head size, referred to as PCKh@0.5. If the position of any predicted joint is within this threshold, the joint prediction succeeds; otherwise, the prediction fails.

### 4.3. Model Design

Figure 5 compares the PCKh@0.5 accuracy results of the state-of-the-art methods with R-HG and QR-HG on the test dataset of MPII. Though the model structure is simplified and the precision of the parameters is reduced to 8-bit, R-HG or QR-HG does not compromise the model generalization capability significantly. Compared to the baseline model HG, the overall joint accuracies from R-HG and QR-HG drop 2.4% and 7.1%, respectively.

### 4.4. Experiment II: Efficiency Performance Evaluation

In this experiment, we evaluate the efficiency performance of the proposed model compared to the baseline model. The model size and image inference time are analyzed. The inference time is measured using the same desktop CPU for all three models. Since the time complexity of calculating ROM is linear in Equation (5), the inference time of the joint estimation algorithm is considered equal to the overall method (joint estimation + ROM measurement) processing time.

As shown in Table 2, the R-HG model is 4 times smaller and 1.7 times faster than HG. The QR-HG model is about 15.5 times smaller and 4.2 times faster than HG. Both R-HG and QR-HG are more cost-efficient than HG and are easier to deploy to low-capacity devices. From the previous experiment in Figure 5, R-HG is 4.6% more accurate than QR-HG in general. So, depending on the accuracy, latency, and memory size requirement, there is a trade-off between choosing R-HG or QR-HG with respect to the effectiveness and efficiency in varying degrees.

### 4.5. Experiment III: ROM Assessment

In this experiment, we compare the measurements obtained by the goniometer with those obtained from the QR-HG processed images to evaluate the ROM accuracy. The mean arc and root mean difference (RMSE) of the ROM are calculated (manual goniometer-obtained measurements and measurements based on the proposed method).

**Figure 6:**
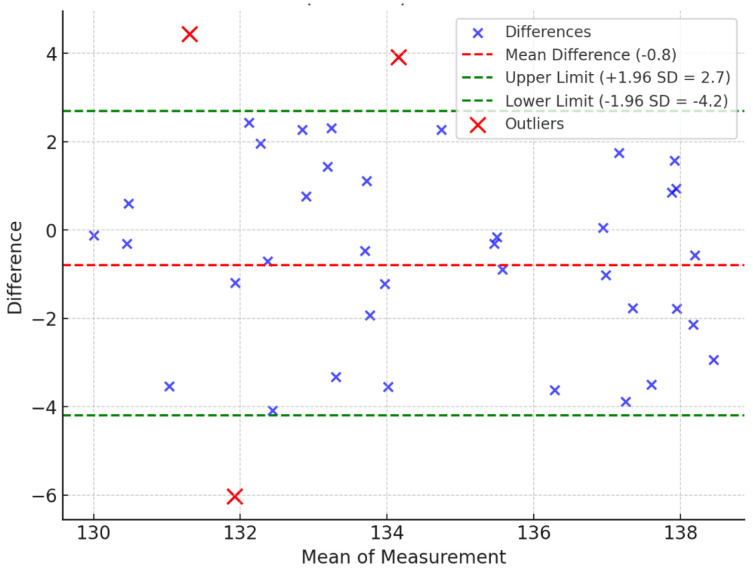
Bland−Altman plot of elbow (R + L) range of motion.

**Figure 7:**
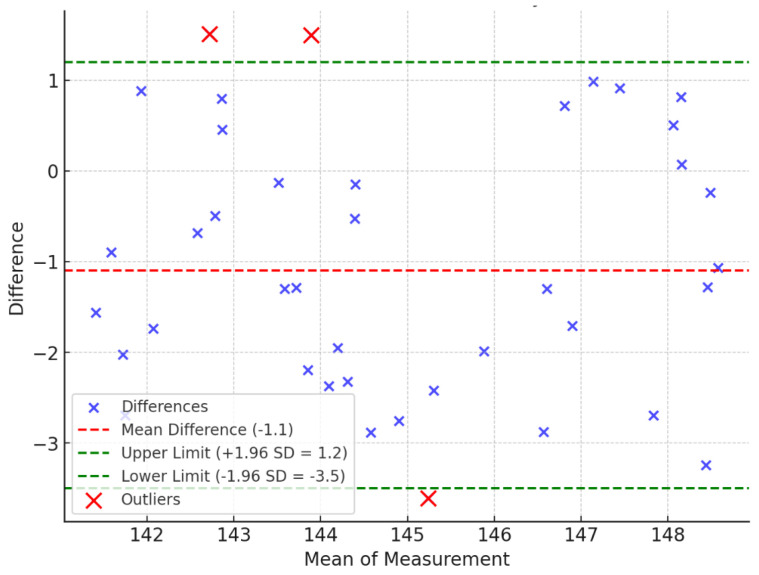
Bland−Altman plot of shoulder (R + L) range of motion.

**Figure 8:**
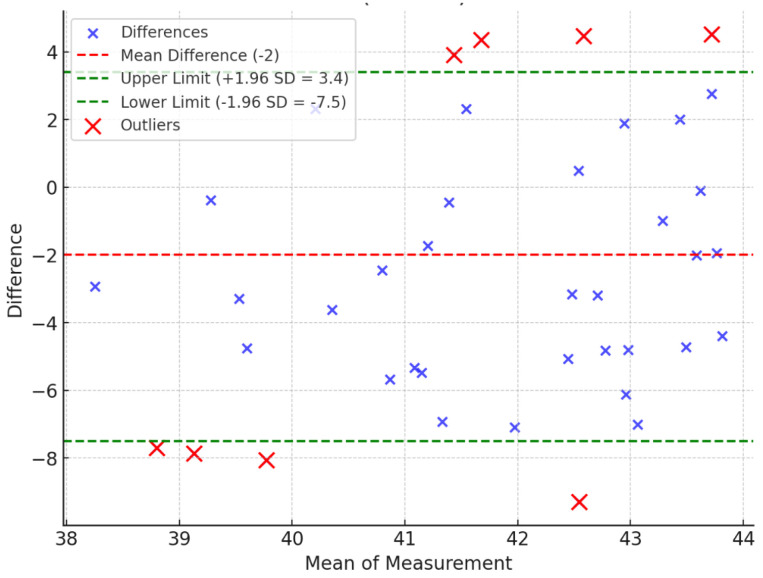
Bland−Altman plot of hip (R + L) range of motion.

The Bland–Altman analysis [42] is used to assess the agreement between two quantitative methods of measurement. The Bland–Altman analysis is to evaluate a bias between the mean differences and to estimate an agreement interval that falls within 95% (+/− SD 1.96) of the differences between the second method, compared to the first one. To check the assumptions of normality of differences and other characteristics, we used a graphical approach as shown in Figure 6, Figure 7 and Figure 8.

The Bland–Altman analysis shows that 37 out of 40 measurements for the elbow, 37 out of 40 for the shoulder, and 32 out of 40 for the hip fall within the 95% confidence interval (CI) (Table 3 and Figure 6, Figure 7 and Figure 8). While the majority of measurements demonstrate a high degree of agreement between the two methods, there are minor fluctuations in the data. These fluctuations may be attributed to variability in human anatomical structures or slight discrepancies in image processing for more complex joint motions. The analysis also reveals that the differences are randomly distributed without any systematic bias, further supporting the reliability of the proposed method.

Overall, the Bland−Altman analysis confirms that the proposed method achieves a high level of accuracy and agreement with traditional goniometer measurements, making it suitable for practical ROM assessments.

### 4.6. Experiment IV: Cost-Effectiveness and Efficiency of Raspberry Pi 4

In this experiment, we discuss the cost-effectiveness of RPi4, taking into consideration equipment and energy costs and evaluating the model’s inference efficiency on the RPi4 platform.

RPi4 equipment cost: The RPi4 setup has a relatively low cost of approximately one hundred USD [43], which encompasses the RPi4 device, a Pi camera, an SD card, and a power adapter. The RPi4 features a Quad-core Cortex-A72 (ARM v8) 64-bit SoC @ 1.8 GHz processor and an LPDDR4-3200 SDRAM memory size of up to 8GB. Considering that a general-purpose computer for healthcare centers or clinics typically comes with a higher price tag, the RPi4 setup encourages medical professionals to explore alternative computing solutions that can satisfy budget constraints [39]. This cost-effective setup helps to promote accessibility and affordability in medical care environments.

RPi4 energy cost: Compared to a general-purpose desktop computer, the RPi4 is a resource-constrained device that benefits from lower power consumption. We measure the power consumption of the RPi4 using a USB wattage meter during both idle and computation-intensive periods. The results revealed that the RPi4 consumes 3.1 watts during idle periods and 7.2 watts while performing inference tasks. In contrast, our CPU-based desktop setup demands approximately 100 to 200 watts of power, making the RPi4 a significantly more energy-efficient option. These substantial power savings not only reduce electricity costs but also enable the device to be operated using battery power, which is particularly advantageous in scenarios where portability or remote access is required.

Accuracy performance of RPi4: In order to ensure consistent results, the proposed RG-HG model has been implemented using the same programming language and deep learning framework on both the RPi4 and the CPU-based desktop platforms. We conduct an evaluation of the model’s inference accuracy on these two platforms to identify any potential disparities. Our findings indicated no significant difference in accuracy between the RPi4 and the CPU-based desktop platforms, yielding a consistent range of motion assessment accuracy across both systems. This consistency allows users to choose either platform for deployment without worrying about potential discrepancies in ROM assessment outcomes.

Efficiency performance of RPi4: To assess the efficiency performance of the RPi4, we conduct an evaluation using the same tasks described in Section 4.1.3, which can be processed on both the RPi4 and desktop platforms. During the evaluation, the RPi4 showcased its ability to process up to 18 static input images per second. To perform a complete ROM assessment, two images are required: one for the starting position and one for the end position. Consequently, this processing capability enables the assessment of 9 functional tasks. This result highlights the RPi4’s potential for real-time processing capabilities and its suitability for use in time-sensitive applications.

By evaluating the four factors in Table 4, we have demonstrated that the RPi4 can perform ROM assessment tasks with the same accuracy as a CPU-based desktop computer while offering superior equipment and energy cost-effectiveness. Given that the RPi4 and camera are readily available consumer components, and their specifications are common in many other IoT hardware platforms [10], we can reasonably expect our proposed RQ-HG ROM assessment model to be compatible with other similar resource-constrained devices. This provides the flexibility to select various platforms for deployment, depending on the users’ needs and available resources.

## 5. Conclusions

This work proposes a deep learning-based model to assess ROM using photographs captured by a 2D RGB camera. We design a compact, quantized CNN model to ensure compatibility with resource-constrained devices, reducing latency and memory requirements. Unlike other HPE or ROM studies, our method focuses on both upper and lower limb joints, enhancing joint estimation accuracy and further reducing latency.

We evaluate the proposed method on datasets collected from six ROM tasks. The results show that our new method achieves a satisfying accuracy in ROM measurement and a high degree of agreement with a goniometer. And our model can run 4.1 times faster and is 15.5 times smaller than one of the most accurate human pose models in our experiment. Additionally, we carry out an experiment on a Raspberry Pi, illustrating that the method can maintain performance while reducing equipment and energy costs. This offers flexibility for platform selection based on users’ needs and resources. This study can serve as a valuable reference for researchers and developers seeking a camera-based, budget-friendly, and high-performance solution for ROM assessment to improve patient care and outcomes in the field of musculoskeletal health.

We acknowledge certain limitations in the current study. First, the number of participants is limited to two healthy individuals, which may not fully demonstrate the generalizability of the method. Although our study serves as a proof of concept by adhering to clinical protocols, we plan to collaborate with medical schools or clinics to collect data from a larger and more diverse group of volunteers. This will allow us to validate the method’s performance across different populations, including elderly individuals, athletes, and rehabilitation patients. Second, the current evaluation relies solely on the MPII dataset due to its relevance and availability of annotated joint data. While the MPII dataset includes approximately 25K images of over 40K individuals performing diverse activities, we plan to extend the evaluation to additional publicly available datasets, such as COCO [44] and Human3.6M [45]. This will help assess the generalizability of the proposed method in a broader range of scenarios and demographic groups.

In future work, we will explore additional improvement strategies that can enhance performance while maintaining the lightweight and fast advantages of our proposed method. We also plan to develop a novel approach that considers data privacy within the ROM assessment model. This method would incorporate privacy-preserving techniques to protect sensitive patient data during the ROM assessment process. Another direction of our future work is to incorporate both active and passive ROM assessment, where images may include multiple individuals, such as the patient and a therapist. The proposed method would involve developing a more advanced object detection and tracking system capable of identifying and distinguishing between the object under test (the patient’s limb) and external sources, such as the therapist’s hands.

## Figures and Tables

**Table 1:** Hourglass model cost-effectiveness.

# Modules	Mean PCKh@0.5	# Param
8	91.9	26M
4	91.4	13M
2	90.5	7M
1	86.4	3M

**Table 2:** Efficiency performance.

	HG	R-HG	QR-HG
Model Size (MB)	376	94	24.2
Inference Time (image/s)	4	6.8	16.7

**Table 3:** Comparison of the ROM measurements obtained on the goniometer and the proposed method using a camera.

Variables	RMSE in ROM (Degree)	Bland–Altman Plots (Within 95% CI)
Elbow (R + L) ROM	3.65	37/40
Shoulder (R + L) ROM	3.21	37/40
Hip (R + L) ROM	4.25	32/40

**Table 4:** ROM assessment tasks on Raspberry Pi 4 vs. CPU-Based Desktop.

Metric	Raspberry Pi 4	CPU-Based Desktop
Equipment Cost	Approximately USD100	Significantly higher
Energy Consumption	3.1 watts (idle) 7.2 watts (inference)	100–200 watts
Accuracy Performance	Comparable to CPU-based desktop	Comparable to RPi4
Efficiency Performance	Up to 18 static images per second	Not specified

## Data Availability

The data presented in this study are available on request from the corresponding author due to privacy.
